# Should Nurses Smoke?

**Published:** 1921-04-09

**Authors:** Gladys M. E. Leigh


*To the Editor of* The Hospital.

### Should Nurses Smoke?

*To the Editor of* The Hospital.

Sir,—Your correspondent "Ierne" fails to differentiate between the hours when a nurse is on duty and off duty. He may as well ask, Is it expedient for a woman doctor to enter a public-house, or for a woman teacher to smoke when taking a class?

A nurse has a perfect right to enter a public-house when she is off duty and out of uniform, provided it is a suitable place to be frequented by an educated and respectable citizen. It is not usual to find women of education and refinement drinking at a public bar. I allow both my nursing and domestic staff to smoke when off duty, and I have so far had no cause to regret the attitude I have adopted.—Yours faithfully, Gladys M. E. Leigh.

